# Comparison of Different Ultrasound Classification Systems of Thyroid Nodules for Identifying Malignant Potential: A Cross-sectional Study

**DOI:** 10.6061/clinics/2021/e2126

**Published:** 2021-01-11

**Authors:** Hua Chen, Jun Ye, Jianming Song, Yuguang You, Weihua Chen, Yanna Liu

**Affiliations:** I Department of Medical imaging and nuclear medicine, Medical College of Nanchang University, Nanchang, Nanchang, Jiangxi, China; II Department of Ultrasonic medicine, The first affiliated hospital of gannan medical university, Ganzhou, Jiangxi, China; III Department of Ultrasonic, The second affiliated hospital of nanchang university, Nanchang, Jiangxi, China

**Keywords:** American Association of Clinical Endocrinologists, American College of Radiology Thyroid Imaging Reporting and Data System, American Thyroid Association, Thyroid Cancer, Ultrasound Examinations

## Abstract

**OBJECTIVE:**

In our organization, it has been necessary in our organization to calculate the risk categories according to the American Thyroid Association (ATA), the American Association of Clinical Endocrinologists/American College of Endocrinology/Associazione Medici Endocrinologi (AACE/ACE/AME), and the American College of Radiology Thyroid Imaging Reporting and Data System (ACR TIRADS) classification systems for each patient, from the year 2019; these are also required to be registered in the database. This creates a barrier to medical collaboration in everyday radiological practice because using multiple rating systems can be confusing for both readers and patients. For the change in routine practice, this study aimed to compare diagnostic parameters of the ATA, AACE/ACE/AME, and ACR TIRADS classification systems for the detection of suspicious thyroid nodule(s) considering the results of fine-needle aspiration cytopathology as the reference standard.

**METHODS:**

Data on ultrasound characteristics (2,000 nodules) and fine-needle aspiration cytopathology (39 nodules) were included in the analysis. The decision making of fine-needle aspiration biopsies was evaluated from the ultrasound characteristics as per the ATA, AACE/ACE/AME, and ACR TIRADS classification systems.

**RESULTS:**

The ATA, AACE/ACE/AME, and ACR TIRADS recommended 26, 32, and 37 nodules for fine-needle aspiration biopsies, respectively. Considering the results of fine-needle aspiration cytopathology as the reference standard, the ATA, AACE/ACE/AME, and ACR TIRADS classification systems had 0.993, 0.996, and 0.998 sensitivity, respectively. The accuracies were 0.641, 0.795, and 0.923, respectively.

**CONCLUSION:**

The ACR TIRADS classification system is less invasive and can identify suspicious nodules more accurately than that of ATA and AACE/ACE/AME.

## INTRODUCTION

The prevalence of thyroid abnormalities varies when reported by different modalities, for example, physical examination or ultrasound of the thyroid gland (1). Physical examination of the thyroid gland suggested 5% suspicious nodules (2), while ultrasound imaging suggested 30-67% suspicious nodules in the same population (3). This indicates that ultrasound imaging over diagnoses thyroid cancer that is found benign in fine-needle aspiration biopsies (1,4). There are several ultrasound classification systems available for diagnosis of suspicious nodules which help fine-needle aspiration biopsies, such as the American Thyroid Association (ATA) (5), the American Association of Clinical Endocrinologists (AACE), American College of Endocrinology (ACE), and Associazione Medici Endocrinologi (AME) (6), and the American College of Radiology Thyroid Imaging Reporting and Data System (ACR TIRADS) (7) because a combination of several suspicious features is required to detect malignancy in a thyroid nodule(s) (8). All three guidelines are generally used in clinical practice for fine-needle aspiration biopsy decision-making (9). The main aim of these guidelines is to reduce unnecessary biopsies and patient harm (10). With the increasing incidence of thyroid nodules, there is a need for the accurate examination of suspicious nodules to avoid overtreatment of benign nodules.

In our organization, it has been necessary to calculate the risk categories according to each of the three guidelines of ultrasound classification systems for each patient, from the year 2019; and these are required to be registered in the database (the institutional protocol). This creates a barrier to medical collaboration in everyday radiological practice because having multiple rating systems in use can be confusing for both readers and patients. The clinical application of differences in the different ultrasound classification systems for the evaluation of risk stratification of thyroid nodules is difficult to define (3). Thus, the development of a universal thyroid nodule ultrasound malignant risk stratification system is necessary.

The objective of this study was to compare the diagnostic parameters of ATA, AACE/ACE/AME, and ACR TIRADS classification systems for the detection of suspicious thyroid nodules for decision making of fine-needle aspiration biopsies, considering the results of fine-needle aspiration cytopathology as the reference standard.

## MATERIALS AND METHODS

### Ethics statement and consent to participate

This study was approved by the Second Affiliated Hospital of Nanchang University review board and the Chinese Society of Clinical Oncology (Protocol no. SHNU15042220 dated April 22, 2020). All enrolled patients provided prior consent for radiology and biopsies (when required).

### Patient population

**Figure 1:**
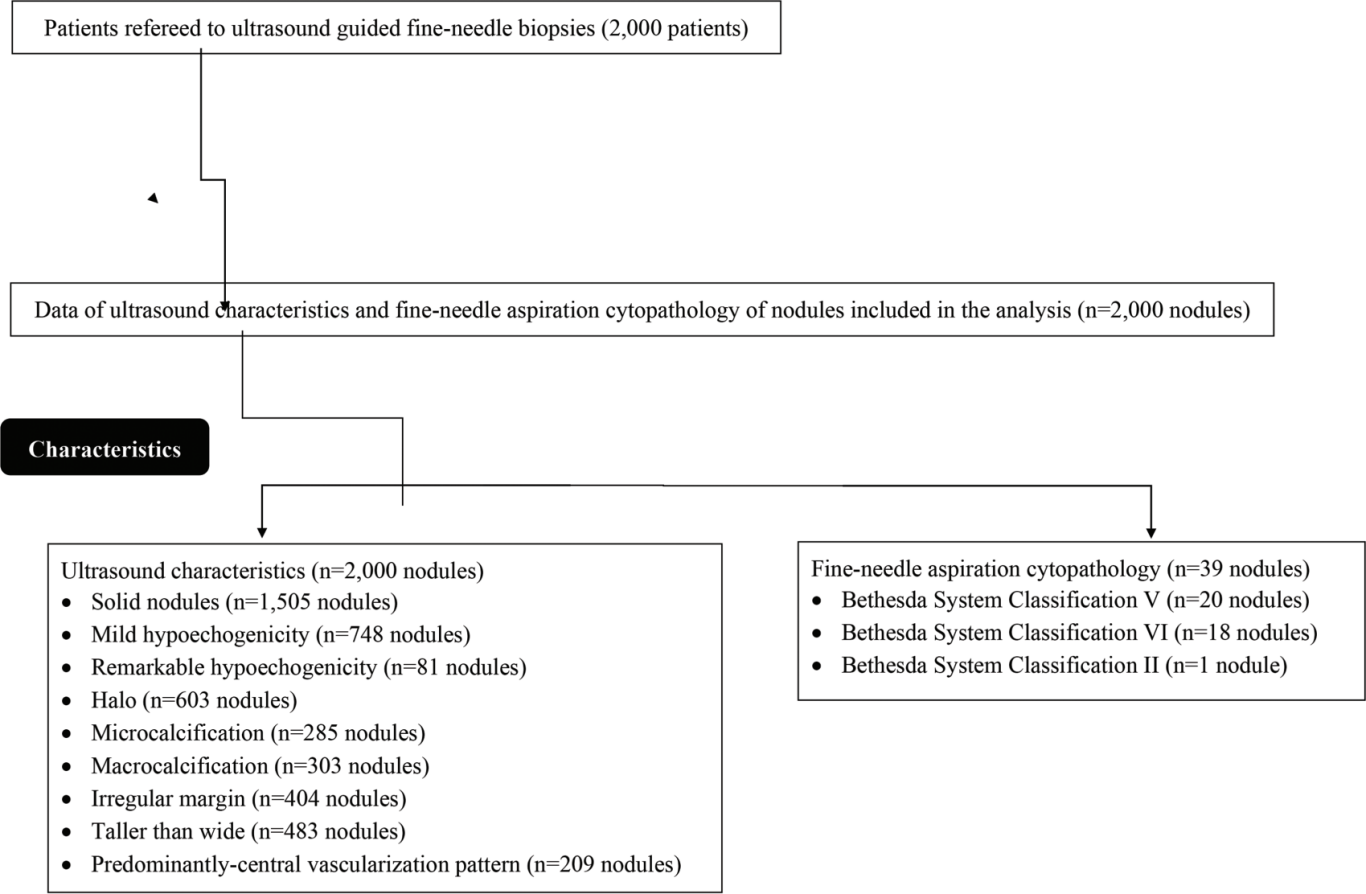
Flow chart of the ultrasound and fine-needle aspiration cytopathology data.

The data of patients with suspected thyroid nodules (palpable neck mass or found incidentally in previous imaging practice) referred for ultrasound-guided fine-needle biopsies were collected and analyzed. The ultrasound features of the patients from the report of the initial interpretation of ultrasound findings were retrospectively collected and their distribution into different ultrasound guidelines was performed. From January 12, 2019 to February 21, 2020, 2,000 patients were referred for ultrasound-guided fine-needle biopsies at the department of radiology and pathology of the Second Affiliated Hospital of Nanchang University, Nanchang, Jiangxi, China and the First Affiliated Hospital of Gannan Medical University, Ganzhou, Jiangxi, China. Among these, 1,785 patients were female and 215 were male. The other demographic and clinical conditions of patients who required admission diagnosis are reported in Table 1. Data on ultrasound characteristics (2,000 nodules) and fine-needle aspiration cytopathology (39 nodules) were included in the analysis (Fig. 1).

### Ultrasound examinations

Ultrasound was performed using an iU22 (Philips Healthcare, Eindhoven, Netherlands) Doppler-ultrasound equipment with 7-15 MHz linear array transducers (10). All ultrasound examinations were performed by ultrasound technologists (a minimum of 5-years of experience in thyroid imaging) of the institutions. In the case of multiple nodules, ultrasound features of biopsied nodules were included in the analysis.

### Image analysis

Before fine-needle aspiration biopsy, the ultrasound characteristics of all nodules were analyzed. Three ultrasound classification systems, the ATA, AACE/ACE/AME, and ACR TIRADS were applied to the ultrasound findings. According to the ATA, nodules were characterized as 1 (benign), 2 (very low suspicion), 3 (low suspicion), 4 (intermediate suspicion), and 5 (high suspicion) (5). According to the AACE/ACE/AME, nodules were characterized as 1 (low-risk nodule), 2 (medium risk nodule), and 3 (high-risk nodule) (6). According to the ACR TIRADS, nodules were characterized as TR 1 (Thyroid Reporting 1; benign), TR 2 (not suspicious), TR 3 (very low suspicious), TR 4 (moderately suspicious), and TR 5 (highly suspicious) (7). Without comet-tail markings, small, punctate hyperechoic foci, and distinct of indeterminate hyperechoic spots were defined as microcalcifications. However, calcifications >1 mm coarse areas were defined as macrocalcifications. Ultrasound images were analyzed by six ultrasound technologists (a minimum of 5-years’ experience in thyroid imaging) of the institutions.

### Decision making of fine-needle aspiration biopsies

According to the ultrasound classification system ATA, fine-needle aspiration biopsies were performed if nodules were regarded to have high or intermediate suspicion and ≥1 cm in the average of the maximum diameter of all planes (Ø), low suspicion and ≥1.5 cm Ø, and very low suspicion and ≥2 cm Ø (5). According to the ultrasound classification systems AACE/ACE/AME, for ≥2 cm Ø and low or medium risk nodules and nodules ≥1.5 cm Ø and high-risk nodules, and ≥0.5 cm Ø nodule with subcapsular or paratracheal lesions characteristics, the fine-needle aspiration biopsies were performed. In addition, in cases of lymph node or extra lymph node spread, family and/or personal history of thyroid carcinoma, radiation exposure, and coexistent suspicious clinical findings, fine-needle aspiration biopsy was performed (6). According to the ACR TIRADS, TR 3 and ≥2.5 cm Ø, TR 4 and ≥1.5 cm Ø, and TR 5 and ≥1 cm Ø nodules were subjected to fine-needle aspiration biopsies (7).

### Fine-needle aspiration cytopathology

Ultrasound guidance was used to perform biopsies using a 23G needle. In cases of multiple nodules, a biopsy of the nodule was performed if it had the highest number of suspicious features on ultrasound findings. A total of 5-6 passages were performed for biopsies. Images of the biopsied nodules were developed in the transverse and longitudinal planes and video clips of the biopsied nodules were developed in at least one plane. The biopsied specimen was sent to the laboratory for cytopathology. The Bethesda System for Reporting Thyroid Cytopathology was used for the interpretation of fine-needle aspiration cytology results (11). Nodules with Bethesda V/VI cytology were considered suspicious. Bethesda II was considered a benign nodule (1). Pathologists (a minimum of 5-years of experiences) of the institutions performed the biopsies and cytopathology.

### Interobserver agreement

General interobserver agreement between six ultrasound technologists was performed using Fleiss’ kappa coefficient (κ) considering κ<0.2: poor agreement, 0.21-0.4: slight agreement, 0.41-0.6: moderate agreement, 0.61-0.8: good agreement, and >0.8: very good agreement (12).

### Diagnostic performance

The ratio of the sum of nodules that were truly recommended for biopsy and truly not recommended for biopsy by the ultrasound classification systems to the total number of nodules included in the analysis was considered as sensitivity. The ratio of nodules truly recommended for biopsy by the ultrasound classification systems to the total number of biopsied nodules was considered to be accuracy.

### Clinical significance

The clinical significance was evaluated for each ultrasound classification system as per Eq. 1 (13):



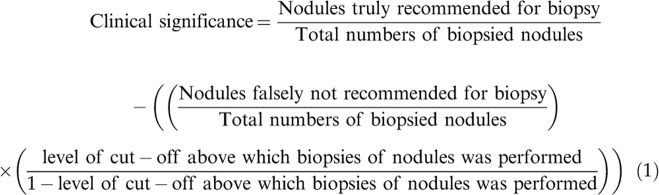



Nodule(s) falsely not recommended for biopsy: Nodule(s) was not recommended for biopsy by the ultrasound classification systems but reported Bethesda V/VI in the cytology.

### Statistical analysis

SPSS V25.0, IBM Corporation, New York, NY, USA was used for statistical analyses. A Chi-square independence test was preferred for a comparison of the proportion of the risk of malignancy to perform fine-needle aspiration biopsies within each ultrasound classification system (1). Fischer’s exact or Chi-square independence tests were performed for statistical analysis of diagnostic parameters. Univariate following multivariate analysis was performed to predict individual risk factors for suspicious nodule(s). All results were considered significant at a 95% confidence level.

## RESULTS

### Ultrasound characteristics and cytological results

Among the nodules enrolled in the study, 1,505 (75%) were solid, 748 (37%) had mild hypoechogenicity, 81 (4%) had remarkable hypoechogenicity, 603 (30%) had halo characteristics, 285 (14%) had microcalcifications, 303 (15%) had macrocalcifications, 404 (20%) had an irregular margin, 483 (24%) had a taller than wide shape, and 209 (10%) had predominantly central vascularization patterns. According to the cytological results, there were 38 suspicious nodules (Bethesda V/VI category) (1.9%) and one (0.05%) was benign (Bethesda II category).

### Risk categories according to different ultrasound classification systems

Higher numbers of high suspicion (*p*<0.0001), high risk (*p*=0.010), and TR 5 (*p*=0.003) nodules were recommended for fine-needle aspiration biopsies by the ATA, AACE/ACE/AME, and ACR TIRADS, respectively. The ATA recommended 26 nodules, the AACE/ACE/AME recommended 32 nodules, and the ACR TIRADS recommended 37 nodules for fine-needle aspiration biopsies. A total of 39 nodules (because of overlapping of systems in a few nodules) were subjected to fine-needle aspiration biopsies following cytopathology (Table 2).

### Interobserver agreement

The ATA (κ=0.31) and AACE/ACE/AME (κ=0.37) ultrasound classification systems had slight interobserver agreement between readers, while the ACR TIRADS (κ=0.41) ultrasound classification system had a moderate interobserver agreement between readers (Table 3).

### Diagnostic performance

Out of 37 nodules recommended by the ACR TIRADS for biopsies, 1 (3%) nodule was falsely recommended. Of the 26 nodules recommended by the ATA for biopsies, 1 (4%) nodule was falsely recommended and out of 32 nodules recommended by the AACE/ACE/AME for biopsies, 1 (3%) nodule was falsely recommended.

Considering the results of fine-needle aspiration cytopathology as a reference standard, the ATA, AACE/ACE/AME, and ACR TIRADS had sensitivities of 0.993, 0.996, and 0.998, respectively. The accuracies were 0.641, 0.795, and 0.923, respectively. The ATA (*p*=0.601), AACE/ACE/AME (*p*=0.692), and ACR TIRADS (*p*=0.809) had the same sensitivities as that of fine-needle aspiration biopsies but the ACR TIRADS alone (*p*=0.239) had the same accuracy as that of fine-needle aspiration biopsy. The other diagnostic parameters for the ATA, AACE/ACE/AME, and ACR TIRADS ultrasound classification systems and their comparisons with the results of fine-needle aspiration biopsies are presented in Table 4.

### Clinical significance

**Figure 2:**
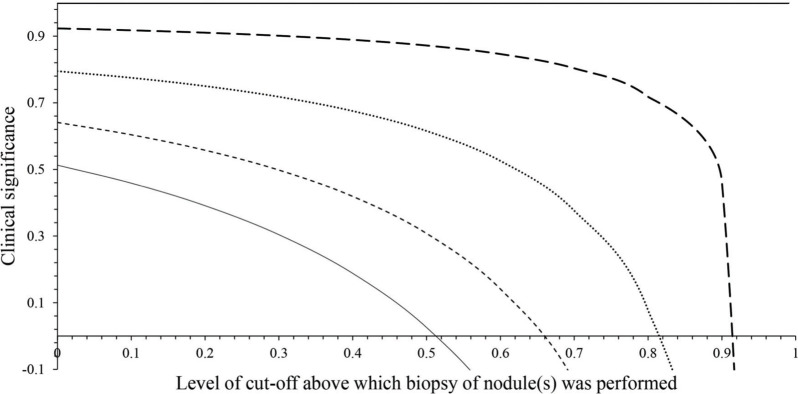
Clinical significance. ATA: the American Thyroid Association, AACE: The American Association of Clinical Endocrinologists, ACE: American College of Endocrinology, AME: Associazione Medici Endocrinologi, ACR TIRADS: the American College of Radiology Thyroid Imaging Reporting and Data System. Cut-off value: Suspiciousness of nodule(s).

The ACR TIRDS had 0-0.914 level of cut-off, AACE/ACE/AME had 0-0.888 level of cut-off, and ATA had 0-0.657 level of cut-off for decision making of fine-needle aspiration biopsies. Cut-off levels above 0.914, 0.888, and 0.657 had the risk of over diagnosis for the ACR TIRDS, AACE/ACE/AME, and ATA systems of ultrasound classification, respectively (Fig. 2).

### Risk of malignancy

Univariate analysis showed that sex, age, history of radiation, family history, thyroid cancer, autoantibodies to thyroid peroxidase, thyroid-stimulating hormone level, and nodule size were associated with suspicious thyroid nodules. Multivariate analysis showed that female sex (*p*=0.021), age less than 45 years (*p*=0.042), previous exposure to radiation (*p*=0.049), and personal history of thyroid cancer (*p*=0.048) were associated with the prevalence of suspicious thyroid nodules (Table 5).

## DISCUSSION

This present study reported that the ACR TIRDS had high accuracy, moderate interobserver agreement, and higher clinical significance than those of the ATA and AACE/ACE/AME for decision making of biopsies. The results of the present study are in agreement with that of other retrospective studies (1,4,9,10) but did not agree with retrospective studies (3,14). The reasons behind the contradictory results of the previous retrospective study included small numbers of nodules and the inclusion of additional images that may change the interpretation of ultrasound examinations. However, the ATA classification cannot detect iso- or hypoechogenic nodules (1). The ACR TIRDS classification system could identify suspicious nodules more accurately than ATA and AACE/ACE/AME.

The ACR TIRDS classification system recommended a lower percentage of unnecessary biopsies than those of ATA and AACE/ACE/AME. These results agreed with previous retrospective (1,4,10,14,15) and prospective (16) studies. The ACR TIRDS (7) has a higher size cut-off for a recommendation of fine-needle aspiration biopsies than the ATA (5) and AACE/ACE/AME (6). The ATA is the most preferred system of classification, but has the risk of over diagnosis (3). This, it can be concluded that the guidelines of the ACR TIRDS ultrasound classification system are less invasive than that of ATA and AACE/ACE/AME.

The ATA and AACE/ACE/AME classification system had slight agreements, while ACR TIRADS had a moderate interobserver agreement. These results were consistent with those of previous retrospective studies (3,17). The interobserver agreement was poor for echogenic foci and substantial for size and microcalcification (17). The ATA classification system is a qualitative while ACR TIRADS is a quantitative method for the stratification of suspicious nodules (3). A standard training for each ultrasound classification system may further improve the interobserver agreement.

This study reported higher numbers of recommended fine-needle biopsies than expected in all ultrasound classification systems, consistent with those of previous retrospective studies (1,3,18). A possible explanation for such disagreements is that the patients included in our study had more confounding risk factors for suspicious nodules. These are risk factors for suspicious nodules, but ultrasound classifications are used for the diagnosis of suspicious nodules, not for detection of the cause of these suspicious nodules.

There are some limitations to this study. First, the retrospective design of this study has its inherent flaws and the results need to be reinforced with prospective studies for a true evaluation of the efficacy. Second, the ATA classification system reported higher specificity than that of ACR TIRADS (14) and AACE/ACE/AME (9), while the ACR TIRADS reported higher specificity than ATA (3,18); however, the specificity data were not reported and discussed. Third, only one nodule per patient was included, which creates bias. Fourth, this study examined three ultrasound categorization systems used in North America to triage thyroid nodules but the difference is not evaluated for other systems in use, for example, Korea, UK-the ultrasound system, where ultrasound categorization systems will be of most interest to a UK-based readership. Fifth, the presented work has a high level of bias because of the substantial-conclusion drawn from only 39 (2%) fine-needle aspiration biopsy results. A larger study may be necessary to evaluate the true risk stratification. Sixth, confounding factors were not evaluated in this study. Although the work shows the correlation of the classification systems (false and true positives), the number of false negatives of each system was not addressed, which is very important for a screening test. Finally, the conclusion that the accuracy of ACR-TIRADS is greater for suspicious nodes is not generalizable because the systems may not always recommend biopsy when the demographic and clinical conditions of patient(s) are suspected. For the general accuracy of the system, it is necessary to include specificity and false negatives.

## CONCLUSIONS

It is difficult to evaluate any single ultrasound classification system that is clinically and statistically significant compared to the other systems. However, the ACR-TIRADS is less invasive, has a moderate interobserver agreement, and can identify suspicious nodules more accurately than the ATA and AACE/ACE/AME systems. We recommend that the Chinese Society of Clinical Oncology needs to adopt the ACR-TIRADS alone for the diagnosis and management of thyroid cancer.

## AUTHOR CONTRIBUTIONS

All authors read and approved the manuscript for publication. Chen H was a project administrator, contributed to visualization, supervision, resources and the literature review of the study. Ye J contributed to conceptualization, resources, validation, the literature review and formal analysis of the study. Song J contributed to the investigation, resources, software, visualization and the literature review of the study. You Y contributed to methodology, resources, the literature review, data curation, and visualization of the study. Chen W contributed to data curation, formal analysis, resources, the literature review and supervision of the study. Liu Ycontributed to the literature review, resources, validation and visualization of the study, draft, review and edited the manuscript for intellectual content. The authors agree to be accountable for all aspects of work ensuring integrity and accuracy.

## Figures and Tables

**Table 1:** Demographic and clinical conditions of patients who were referred for ultrasound-guided fine-needle biopsies.

Characteristics		Patients or value
Data of patients included in the analysis		2,000
Sex	Male	215 (11)
	Female	1,785 (89)
Age (years)	Minimum	27
	Maximum	74
	Mean±SD	53.52±9.17
Body mass index (kg/m^2^)		24.91±1.95
Ethnicity	Han Chinese	1,839 (92)
	Mongolian	142 (7)
	Tibetan	19 (1)
Previous exposure to radiation		55 (3)
Personal history of thyroid cancer		84 (4)
Family history of thyroid cancer		107 (5)
Diabetes		295 (15)
Autoantibodies to thyroid peroxidase positive		612 (31)
Higher thyroid-stimulating hormone level (>2.5 mU/L)		589 (29)
Nodule size according to ultrasound finding (cm)	Minimum	0.61
	Maximum	6.12
	Mean±SD	2.55±0.85

Constant data are presented as frequency (percentage) and continuous data are presented as mean±SD.

**Table 2:** Risk categories according to different ultrasound classification systems.

Characteristics	Categorized nodules	Recommended for fine-needle aspiration biopsies	Comparisons between categorized nodules and nodules recommended for fine-needle aspiration biopsies
ATA			
Data of nodules included in the analysis	2,000	26	*p*-value
Benign	22 (1)	0 (0)	<0.0001
Very low suspicion	42 (2)	2 (8)	
Low suspicion	1,151 (58)	4 (15)	
Intermediate suspicion	408 (20)	7 (27)	
High suspicion	377 (19)	13 (50)	
AACE/ACE/AME			
Data of nodules included in the analysis	2,000	32	*p*-value
Low risk nodule	42 (2)	2 (6)	0.010
Medium risk nodule	994 (50)	8 (25)	
High-risk nodule	964 (48)	22 (69)	
ACR TIRADS			
Data of nodules included in the analysis	2,000	37	*p*-value
TR 1	61 (3)	0 (0)	0.003
TR 2	142 (7)	2 (5)	
TR 3	531 (27)	11 (30)	
TR 4	918 (46)	9 (24)	
TR 5	348 (17)	15 (41)	

Data are demonstrated as frequency (percentage).

Fischer exact test was used for statistical analysis.

*p*<0.05 was considered significant.

ATA: American Thyroid Association, AACE: The American Association of Clinical Endocrinologists, ACE: American College of Endocrinology, AME: Associazione Medici Endocrinologi, ACR TIRADS: the American College of Radiology Thyroid Imaging Reporting and Data System.

**Table 3:** Interobserver agreement for ultrasound classification systems.

Ultrasound classification systems	Fleiss’ kappa coefficient (κ)
Numbers of readers	06
ATA	0.31
AACE/ACE/AME	0.37
ACR TIRADS	0.41

κ<0.2: poor agreement, 0.21-0.4: slight agreement, 0.41-0.6: moderate agreement, 0.61-0.8: good agreement, and >0.8: very good agreement.

ATA: American Thyroid Association, AACE: The American Association of Clinical Endocrinologists, ACE: American College of Endocrinology, AME: Associazione Medici Endocrinologi, ACR TIRADS: the American College of Radiology Thyroid Imaging Reporting and Data System.

**Table 4:** Diagnostic performance of ultrasound classification systems.

Characters	Fine-needle aspiration biopsies	ATA		AACE/ACE/AME		ACR TIRADS	
Nodules included in analysis	39	2,000	** *p*-value	2,000	** *p-value*	2,000	** *p-value*
Nodules truly recommended for biopsy	39 (100)	25 (1.25)	<0.0001	31 (1.55)	<0.0001	36 (1.8)	<0.0001
Nodules truly not recommended for biopsy	0 (0)	1,961 (98.05)	<0.0001	1,961 (98.05)	<0.0001	1,961 (98.05)	<0.0001
Nodules falsely recommended for biopsy	0 (0)	1 (0.05)*	0.889	1 (0.05)*	0.889	1 (0.05)*	0.889
Nodules falsely not recommended for biopsy	0 (0)	13 (0.65)*	0.614	7 (0.35)*	0.711	2 (0.1)*	0.843
Sensitivity	1	0.993*	0.601	0.996*	0.692	0.998*	0.809
Accuracy	1	0.641	0.001	0.795	0.009	0.923*	0.239

Data are demonstrated as frequency (percentage).

** Respect to the results of fine-needle aspiration biopsies.

Fischer exact or Chi-square independence test was used for statistical analysis.

*p*<0.05 was considered significant.

* Insignificant difference with respect to the results of fine-needle aspiration biopsies.

ATA: American Thyroid Association, AACE: The American Association of Clinical Endocrinologists, ACE: American College of Endocrinology, AME: Associazione Medici Endocrinologi, ACR TIRADS: the American College of Radiology Thyroid Imaging Reporting and Data System.

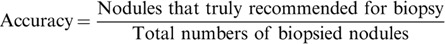

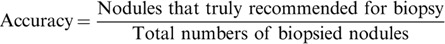

**Table 5:** Demographic and clinical conditions of patients associated with suspicious nodule.

Data of patients included in the analysis	39		
Characteristics	Odd ratio	95% confidence limit	*p*-value
Sex (female* *vs*. male)	1.212	0.658-0.954	0.021
Age (<45 years* *vs*. ≥45 years)	1.113	0.721-0.913	0.042
Previous exposure of radiation (yes* *vs*. no)	1.032	0.711-0.923	0.049
Personal history of thyroid cancer (yes* *vs*. no)	1.045	0.722-0.941	0.048
Family history of thyroid cancer (yes *vs*. no)	0.989	0.756-0.895	0.052
Autoantibodies to thyroid peroxidase (positive *vs*. negative)	0.976	0.721-0.895	0.058
Thyroid-stimulating hormone level (>2.5 mU/L *vs*. ≤2.5 mU/L)	0.984	0.776-0.852	0.053
Nodule size according to ultrasound finding (<2 cm *vs*. ≥2 cm)	0.683	0.562-0.796	0.061

Multivariate analysis.

Data of patients not performed for biopsies were considered as a reference standard.

Odd ratio>1 and *p*<0.05 were considered significant.

* Significant parameter associated with suspicious nodules.
